# A Narrative Inquiry of East Asian Parents and Mental Health in Canada: Critical Openings for Anti-Racism Strategies in Knowledge Translation

**DOI:** 10.1177/08445621251322552

**Published:** 2025-03-17

**Authors:** Samantha Louie-Poon, Solina Richter, Diane Kunyk, Shannon D. Scott

**Affiliations:** 1 Faculty of Nursing, 3158 University of Alberta, Edmonton, AB, Canada; 2 College of Nursing, 7235 University of Saskatchewan, Saskatoon, SK, Canada

**Keywords:** East Asian, knowledge translation, children and adolescent mental health, anti-racism, narrative research

## Abstract

**Background:**

Anti-Asian racism is linked with adverse mental health conditions in young East Asian populations. There is a need to explore how to develop mental health resources for East Asian parents, yet minimal research explores anti-racism strategies for this work.

**Purpose:**

The objectives were to: open a critical dialogue for developing anti-racism strategies for mental health knowledge translation (KT) resource development, and explore complexities with engaging East Asian parents when developing KT resources.

**Methods:**

A narrative inquiry was conducted to collect East Asian parent stories on anti-racism strategies and mental health. East Asian parents across Canada engaged in semi-structured interviews between August to October 2022. Dialogic/performance analysis was used to inductively analyze the data. Findings: Three composite counter-narratives emerged from the data: 1) Storying issues of access within child mental health KT; 2) Seeking understanding and solidarity for the East Asian identity and story; 3) Unlearning, breaking barriers, and storying resistance. The composite narratives wove together seven storylines: a) availability and affordability, b) language and vocabulary barriers, c) lack of representation, d) issues of representation: power and whiteness, e) East Asian standpoint epistemology, f) breaking cycles, g) culture as a source of strength.

**Conclusion:**

The findings highlighted the complexities of engaging East Asian parents and recommended the need for an East Asian standpoint epistemology when developing child mental health KT resources and counter-spaces as a way to facilitate the centrality of East Asian standpoint epistemologies. These anti-racism strategies may promote solidarity for shared experiences beyond the white gaze and spaces.

The coronavirus (COVID-19) pandemic emphasized how racism towards Asian populations continues to exist within the foundation of Canadian society. Asian communities in Canada self-reported incidences of discrimination during the COVID-19 pandemic including physical force/aggression, being coughed at/spat on, and verbal harassment (Chinese Canadian National Council Toronto Chapter, 2022). While Asian communities in Canada have shared some of these collective experiences of racism, Asian populations include multiple ethnicities, cultures, and histories that influence their experiences of racism in society. According to the Chinese Canadian National Council Toronto Chapter (2022), more than half of the victims’ self-reports of anti-Asian hate crimes during the COVID-19 pandemic were from East Asian communities. In this study, we define East Asian as those who self-identify as Chinese, Korean, Japanese, or Taiwanese ethnicity, including individuals self-identifying from autonomous regions, for example, under Chinese sovereignty. With this backdrop, it is critical to address the unique experiences of racism from the East Asian perspective in Canada.

The COVID-19 pandemic has negatively impacted East Asian communities including their mental health. Specifically, the surge in COVID-19-related anti-Asian racism has intensified mental health conditions (e.g., mental health symptoms, mental health disorders) on East Asian children (Cheah et al., 2020; Johnson et al., 2021; Wu et al., 2021). We are broadly defining mental health conditions to include mental disorders and other mental states linked with distress or impaired in functioning (World Health Organization, 2022). Experiences of anti-Asian racism have been linked with trauma-based mental injury, such as racial trauma, for Asian families (Michel, 2019; Wu et al., 2021). A North American study found that in Chinese children and youth, COVID-19 racial discrimination and Sinophobia (fearing Chinese diasporas) were positively associated with anxiety symptoms and internalizing problems (Cheah et al., 2020). These findings are supported by other studies collecting victims’ self-reports of hate crimes revealing that East Asian diasporas in Canada made up more than half of reports in 2021, with 71% of all reported harm causing mental distress or emotional harm (Chinese Canadian National Council Toronto Chapter, 2022).

Previous studies have documented that racial discrimination towards young East Asian populations is related to increased mental disorders (e.g., anxiety) (Juang & Alvarez, 2010), heightened adverse mental health traits (e.g., somatization) (Juang & Alvarez, 2010; Rivas-Drake et al., 2008), and negative psychological adjustment (e.g., self-esteem) (Niwa et al., 2014; Rivas-Drake et al., 2008; Shrake & Rhee, 2004). Despite the past and present demands to address the mental health of East Asian children, evidence suggests that a scarcity of East Asian parents seek mental health services for their children (Liu et al., 2020; Nguyen-Truong et al., 2021).

The underutilization of mental health resources has previously been framed within an individualistic and culturalist understanding, such as pointing to East Asian cultures or individual behaviors, as the source or reason for the underutilization of resources and services (Augsberger et al., 2015; Hails et al., 2018; Kim et al., 2018, 2021; Ng, 1997). We contend there are more comprehensive approaches that explore the interplay between structural barriers and healthcare decision making processes. These approaches, for example, may focus on the impact of racism and East Asian parents’ perception and utilization of mental health resources for their children (Louie-Poon et al., 2022). There is a need to shift the focus to explore the impact of racism within child mental health resources and how racism influences the access and utilization of these resources for East Asian parents.

Critical analyses of racism have recently emerged within knowledge translation (KT) scholarship, a field dedicated to understanding how to move evidence-based research into the hands of end-users (Graham et al., 2006; Straus et al., 2013a). Recent literature calls into question the dominating Western-centric epistemological underpinnings of KT to highlight the power dynamics within KT practices which require further attention (Crosschild et al., 2021). Others within the field have taken a specific focus on anti-racism in the field of implementation science by centering lived experiences, interrogating what ‘counts’ as evidence, and facilitating reflection and accountability (Shelton, Adsul, and Oh, 2021; Shelton, Adsul, Oh, Moise, et al., 2021). Similarly, others have embedded intersectionality frameworks within KT research (Etherington et al., 2020; Sibley et al., 2022), illuminating the tensions and divides of utilizing an intersectionality lens within a KT framework (Kelly et al., 2021). For example, scholars have highlighted how many KT theories, models, and frameworks decontextualize behavior change, use deductive application of theoretical frameworks and predetermined coding structures (e.g., Consolidated Framework for Implementation Research) lacking factors like racism that affect change, and exclude qualitative or lived experiences as evidence (Allen et al., 2021; Kelly et al., 2021). While tensions between the field of KT and intersectionality have been documented, the shift towards integrated knowledge translation (iKT) aligns with an intersectionality framework given the focus on lived experiences of end-users (Kelly et al., 2021). The iKT approach is defined by the process in which researchers share power with knowledge users to identify research problems and implement outputs generated from the research (Beckett et al., 2018; Nguyen et al., 2020). In the development of patient decision aids specifically, tenets of iKT suggest the use of power sharing by inviting patient partners in the development and dissemination of such resources (Brouwers et al., 2013; Straus et al., 2013b). The iKT approach presupposes that research outputs are more likely to produce positive results (e.g., knowledge users utilizing KT resources) when knowledge users are actively involved in the research process (Nguyen et al., 2020; Sibley et al., 2022; Straus et al., 2013b).

These approaches have the potential to produce anti-racist KT interventions as the focus remains on the unique community needs. While this emerging research is promising, there remains minimal empirical research that explores racial positioning within the context of white supremacy and KT research when working with East Asian populations. The emerging body of KT theories, models, and frameworks promote collaborative, user-centered KT approaches (Baumann & Cabassa, 2020; Jenkins et al., 2016; Kitson et al., 2013; Shelton, Adsul, Oh, Moise, et al., 2021). KT process models, including the Co-KT Framework (Kitson et al., 2013) and the CollaboraKTion Framework (Jenkins et al., 2016), are centered within community environments, underscore the shifting nature of collaborative work, and acknowledge the diverse contexts within which KT interventions occur (Jenkins et al., 2016). While these KT process models offer progress and insights into community-based and user-centered designs, critically reflecting how researchers may work to confront and dismantle racism in KT remains outside the scope of these approaches. Recently, calls within the field of implementation sciences and KT have specifically focused on inequities and racism through case study and dialogue approaches (Baumann & Cabassa, 2020; Crosschild et al., 2021; Shelton, Adsul, Oh, Moise, et al., 2021). Yet, there remains limited empirical research exploring racism and KT research. These contributions provide a critical foundation for anti-racism in KT, and the groundwork to begin conducting empirical research.

Gaining a deeper understanding of the specific complexities of how racism shapes East Asian perspectives is critical to further illuminate how KT resources may be changed based on their unique contexts (Woodward et al., 2021). In the absence of anti-racism strategies within the KT process, East Asian parents’ perceived acceptability of prospective child mental health KT tools, their engagement and utilization of these patient decision aids, may be limited (Sekhon et al., 2017). This poses concerns for the nursing profession given nursing's critical role in developing and facilitating the use of patient decision aids (Silvia et al., 2008; Tate et al., 2021).

Nurses and other healthcare professionals have a collective moral imperative to advocate for social justice within our practice, inclusive of our research processes, to uphold our ethical principles (Louie-Poon et al., 2021). Uncovering and redressing racism in KT research processes is one such opportunity for nursing to engage and commit towards its social justice mandate. Moreover, shedding critical light on racism in KT processes may open avenues for anti-racism nursing practices in the future, including alternative possibilities for patient decision aids that may be developed and utilized while balancing calls for acceptability and inclusivity. Yet, the area of anti-racism and KT remains undertheorized in the Canadian context, and even more significantly underexamined within the context of child mental health KT for East Asian populations. There is a need for critical dialogue and reflection prior to seeking solutions for other areas of nursing practice, such as policymaking, to prevent (in)advertently reinforcing white supremacy ideologies (Louie-Poon, 2021). We are particularly drawn to Dillard-Wright's (2022) articulation of a radical imagination in the nursing context that reckons with the boundaries of oppressive normativities, including whiteness, of the discipline by speculating on other possibilities for the future of nursing. In this line of thinking, we purport it is critical to first engage in this space of radical imagination, through speculation and critical dialogue, within KT research processes prior to moving towards urgent solutions for other areas of nursing practice.

Given the need to support mental health topics with East Asian communities from an anti-racist perspective, qualitative approaches that seek the perspectives of racialized populations may deepen our understanding and promote a space of critical reflection and radical imagination. Particularly, it may support our efforts to gain an in-depth perspective on the complex, nuanced, and specific conditions of communities with lived experiences without inadvertently reinforcing inequities (Shelton, Adsul, & Oh, 2021; Shelton, Adsul, Oh, Moise, et al., 2021). A narrative study into the stories of East Asian populations was guided by the research question: *How can researchers utilize anti-racism principles when developing mental health KT resources for East Asian children*? The objectives of this study were to: a) open a critical dialogue for developing novel anti-racism strategies for future mental health KT resource development, and b) explore complexities with engaging East Asian parents in a research environment when developing KT resources.

## Methods

The current study was part of a larger narrative inquiry study exploring East Asian parents’ narratives of racism, anti-racism, and mental health. This paper reports on East Asian parent narratives on anti-racism strategies for child mental health resources. This study was framed by Critical Race Theory (CRT), aligning with Riessman's (2008) assertion that narrative methods have the capacity to promote social justice when systemically oppressed communities retell their stories to a larger audience. When rooted in CRT, the collective counter-narratives told by participants serve to challenge the status quo (Clark & Saleh, 2019).

### Sample

Given that East Asian populations have shared (e.g., Asian identity) and unique (e.g., multiple ethnicities within East Asian communities) experiences of racism at the intersection of their identities, purposive sampling was undertaken to identify participants from a diversity of East Asian ethnicities, with varying numbers of children, self-identifying as being born in Canada, and self-identifying as immigrating to Canada. CRT challenges the oversimplification of the global human experience by promoting an exploration into the individual context of communities seeking liberation (Delgado & Stefancic, 2013, 2017). However, strategic essentialism of minoritized communities based on shared identity, that is, temporarily utilizing essentialist positions based on identities such as ethnicity or race, is a strategy to advocate towards change (Pande, 2017; Spivak, 1990). Therefore, this study utilizes guidance from CRT (Delgado & Stefancic, 2013, 2017) and Spivak (1990) to document the self-reported ethnicities of participants to avoid homogenizing the diversity existing East Asian communities, while underscoring shared experiences from systemic racism through a collective East Asian voice that is unique from the pan-Asian or broader racialized experience.

Participants were recruited through a combination of online social media strategies and community word-of-mouth. Digital graphic materials were shared using two strategies: a) distributed to the social media platforms (i.e., Facebook, Twitter, Instagram) of child health research programs, and b) circulated to the social media platforms (i.e., Facebook, Instagram) of Canadian community organizations with a broad focus on anti-racism. Participants were included if self-identified as East Asian (i.e., Chinese, Japanese, Korean, Taiwanese), lived in Canada, were a parent or a caregiver of a child of East Asian descent, fluent in English, over 18 years old, had access to Zoom, and self-identified having experience(s) of racism.

### Data Collection

Riessman's (2008) narrative interviewing guided the data collection methods. A total of 16 virtual (i.e., Zoom) interviews were conducted with 8 participants between August 2022 to October 2022. Sixteen interviews were conducted by the first author (SLP) for consistency and lasted between 50 to 60 min. Two interviews were conducted with each participant. The purpose of conducting multiple interviews with a smaller sample size was to develop a comprehensive narrative account (Riessman, 2008). The interview process followed the “participants down their trails” (Riessman, 2008, p. 24). Interview questions began with broad, open-ended questions that focused on a specific time, event, or place to evoke a detailed narrative account (e.g., Can you tell me about a time you experienced racism in the context of mental health?). Follow-up questions were asked to prompt participants to provide further details (e.g., What happened in this moment? Why does this moment stand out?). Reflexive field notes were documented after each interview to record impressions and contexts. Interviews were audio recorded, professionally transcribed verbatim, and cleaned for accuracy.

### Data Analysis

To inductively analyze the data, dialogic/performance analysis was undertaken to answer how the context entered into the storying experience of participants’ oral narratives (Riessman, 2008). CRT was used as an entry-point for reading and interpreting the interview text. For example, the research team paid particular attention to how contexts of racism are inscribed overtly and covertly within the text to uncover neglected histories and to move away from neutrality in racial struggles (Delgado & Stefancic, 2013, 2017). This included using whiteness as a foundation for the analysis. Whiteness is a socially and politically constructed system whereby white cultural practices are normative, resulting in a racial hierarchy that oppresses those considered as ‘other’ or non-white (Delgado & Stefancic, 2013, 2017). Additionally, the tenet of counter-narratives in CRT recognizes the importance of utilizing the standpoint of East Asian populations (Delgado & Stefancic, 2013, 2017), and subsequent literature supporting notions of standpoint, including components of feminist standpoint theory (Harding, 1997), further informed data analysis. The first author brought their positioned identities as an East Asian person into the interviewing process and interpretation of the data (Riessman, 2008), while iteratively sharing reflections with the research team. A close reading across all participants’ first interviews was iteratively conducted (SLP) while being situated within the aforementioned contexts. The first author determined the beginning and end of narrative blocks. Narrative blocks describe an extended account in the interview transcript and are used to preserve the participants’ stories as the unit of analysis (Riessman, 2008). Preliminary storylines were coded in NVivo software by grouping narrative blocks with similarities and parallels. Decisions, reflections, and refinement of preliminary storylines were discussed with the research team. The first author then conducted a close reading of all participants’ second interviews and re-read participants’ interviews chronologically (i.e., participant 1 interview 1, participant 1 interview 2). Storylines were redefined based on the new data and discussed with the research team.

Composite narratives were developed to present the findings following the guidance developed by Willis (2018b). The composite narratives are a collection of participant verbatim quotes from the narrative blocks grouped within each storyline. Each composite narrative weaves together the voices of all eight participants. Given that narrative data develops rich, detailed accounts of individual stories, this may pose safety concerns when presenting data, particularly in areas where participants experience systemic oppression (Patton & Catching, 2009; Saleh et al., 2023). Therefore, constructing a composite narrative offers a strategy to re-tell a collective story while illuminating detailed accounts of individual stories that protect the identities of systemically vulnerable populations (Solórzano & Yosso, 2002; Willis, 2018a, 2018b).

### Rigour

Reflexive notes were maintained throughout the study. Prolonged engagement with the participants was maintained through multiple in-depth interviews with the same interviewer and participants. A thick, rich description of the research findings were supported by including direct participant quotations (i.e., composite narratives) to support the storylines. Investigator triangulation was utilized by holding meetings to debrief and discuss each interview, the methodological and analytical process, and enhance and confirm findings (Carter et al., 2014). A detailed audit trail was used to document all analytical and process decisions (Denzin & Lincoln, 2018). Critical self-reflexivity was practiced by analyzing our philosophical assumptions, acknowledging our fluid insider-outsider identities, and how power and positionality are inherently embedded within the research process (Crosschild et al., 2021; Mills & Lee, 2015).

### Ethics

Ethics approval was obtained from the University of Alberta Research Ethics Board. Informed consent was obtained at the start of the first interview, and each subsequent interview. Given the topic of racism, participants may experience further discrimination if confidentiality and privacy were not maintained. Therefore, to ensure the safety of all participants, all identifying information, except participant ethnicities, immigrant status, and number of children, were removed from the composite narratives and reporting of data. All participants were provided a list of external resources given the nature of the topic.

## Results

### Participants

A total of 8 participants were included in this study. Of the 8 participants, four self-identified as Chinese ethnicity, three as Japanese ethnicity, and one as Taiwanese ethnicity. Of the Chinese participants, three self-identified as being born in Canada and one self-identified as immigrating to Canada. Of the Japanese participants, one self-identified as being born in Canada and two self-identified as immigrating to Canada. The participants of Taiwanese ethnicity self-identified as immigrating to Canada. Of the 8 participants, they ranged from having 1 to 3 children.

### Storylines

The following sections report on the collective-counter narratives from the 8 parent participants. Each counter-narrative includes verbatim quotes from all participant interviews woven together to form a collective voice. Narratives were grouped and re-storied as three composite narratives with parallel storylines. The storylines within each composite narrative are outlined in Table 1. See Supplemental File A for selected participant quotes for each storyline.

**Table 1.** Composite narratives and storylines.

Composite Narratives	Storylines
**Composite narrative 1:**	Availability and affordability
*Storying issues of access within child mental health KT*	Language and vocabulary* (i.e., terminologies) barriers
	Lack of representation
**Composite narrative 2:**	Issues of representation: power and whiteness
*Seeking understanding and solidarity for the East Asian story*	East Asian standpoint epistemology
**Composite narrative 3:**	Breaking cycles
*Unlearning, breaking barriers, and storying resistance*	Culture as a source of strength

* Note: Historically, the term used to describe this concept was labeled as ‘mental health literacy’. In this study, we are purposefully using the term ‘vocabulary’ to avoid further perpetuating ableism and racism through our language. To state that East Asian peoples are ‘illiterate’ in mental health words due to their culture, unconsciously promotes whiteness and normalizes the dominant Western view of mental health as the correct way of viewing health and wellbeing.

#### Composite Narrative 1: Storying Issues of Access Within Child Mental Health KT

Composite narrative one reports on three storylines: *availability and affordability*; *language and vocabulary (terminologies) barriers*; and *lack of representation*. *Availability and affordability* highlight easily accessible (e.g., in schools) and free of cost resources. Participants describe *language and vocabulary barriers* as the lack of language options, difficulties to arrange interpreters and translators, and the potential for having limited knowledge of the terminologies to discuss mental health matters. *Lack of representation* describes the participants’ desire for representation of East Asian experiences in child mental health resources.

If my children needed mental healthcare, I wouldn’t even know where to look (1). I have no idea because this is never brought up (2). I think that mental health resources should be available in public schools (7). If mental healthcare were more affordable, it would be more accessible to East Asian communities (4). When I previously was exploring different mental health options, the main thing was that I needed it to not cost anything because I couldn’t afford anything (5).

For healthcare providers, they are in the system, and it is easier to navigate than someone who is completely unfamiliar with healthcare. From the inside, they know which resources they could access. But most people don’t have that kind of advocacy within the healthcare system. In the healthcare system, nothing is done in a systematic way in terms of cultural and racial issues. It is always discussed in a fairly superficial way (2). There is no centralized system for East Asian people to go to (3).

I see how mental health resources are presented in mostly just English and French. If people don’t have those two languages, it is awful. How do they access that information (6)? People who do not have English skills have a harder time making it known what their issues are exactly (2). The language barrier can have some effects (3), and is an integral part of what East Asian people might experience (6). Interpreters and translators are not always there and it is a bit of trouble to arrange (2). I can do the research, but if there are no websites that translate back to the language I prefer to operate in, it is very frustrating. I don’t have the energy supply to really look for multiple resources or services that offer translation. Google translate has word limits and limits on how much can be translated. It is taxing to use, and even though these are options, somebody who is less digital savvy, in a new environment, and in more social economic stress will have a shorter of a fuse or attention span to go through translating word by word, phrase by phrase (8).

With mental health, it is so important to be able to be specific when I am describing my child's concerns and conditions. Even for one problem, there might be a checklist of ten different things that I have to be asked about. If my child's concern is very specific, very detailed, and the more I am able to describe and express that, I think the better the chances are that I will know appropriate services to treat their problem. Whereas, if I can’t describe what they are feeling in English and the less detail I am able to put out, then the much more difficult it is to try and get help for the problem (2). So, making communication material more available in different languages can make things more equitable for other people (6).

There is also a language barrier in the sense that I don’t even know the language or the vocabulary to talk about mental health; to know what to search. In the household where I grew up, we didn’t talk about mental health. A lot of my fellow friends with the same background don’t have the vocabulary to talk about mental health. This is compounded when people are new to Canada and can’t speak English well (8).

The language barrier has a lot to do with having representation and a sense of community (6). The lack of suitable East Asian mental health experts is a barrier. No matter how great a white person is, they cannot understand (3). A white person would not know any part of the cultural or racial challenges (5). It would feel more relatable if there was a sheet or resource with certain East Asian experiences. Right now, a family member is in desperate need of some mental health help. But they will not seek it out despite having all the current resources available to them (3). So, it would help to have more representation and have more options available with a deep understanding of our culture and background. When there is more representation, it is a sense of belonging. It is almost like visceral—I feel my body relax. It is a comfort (4).

To access mental health resources for my children, the first step would be to reach out to people I know that I feel safe with, who have similar experiences, similar backgrounds. Finding the right mental health resource is so important. It could do damage if I picked the wrong one, like a resource that doesn’t understand the dynamics of the East Asian experience. I want somewhere, someone, a service, that I feel comfortable with. That I feel safe with. I don’t want to just pick one. I would really look for one that meets my needs; that understands East Asian people. (1). To get more representation, would bring more access to communities where maybe English is the second language and where there is a cultural barrier. Parents and children would feel assured that it is safe and secure. That kind of access would be a better outcome for everybody (6).

#### Composite Narrative 2: Seeking Understanding and Solidarity for the East Asian Story

Composite narrative two reports on storylines: *issues of representation: power and whiteness*, and *East Asian standpoint epistemology*. Through *issues of representation: power and whiteness*, participants move beyond identifying the lack of representation; rather, identify how child mental health resources are predominately developed without an understanding of their East Asian identity and highlight the harms of maintaining whiteness. *East Asian standpoint epistemology* stories the indescribable components of the East Asian identity. Participants identify that these narratives, often told at the intersection of multiple axes of oppression, cannot be fully understood by those without lived experiences.

There are so many mental health studies and research that have a very predominately white audience (6), and I battle what to say. I don’t know what to say. I know if I try to explain (to white people) that they should be happy the next generation of East Asian people are going to grow up in a world where (we) see people who look like (us), they wouldn’t understand (…) because their perspective has always been there (3).

This matters because of the additional challenges of dealing with race-related issues is lost. It is the experience from non-East Asian or non-ethnically diverse audiences (6). It is about knowing the indescribable, non-language understanding of how things are. The unspoken rules. The unwritten background. I can try to explain this. And others can try to understand it but they will only know to the degree of the stereotypical ideas; the things that they see on TV (7). When I am surrounded by an entire circle of people who are East Asian, I am able to relate and be comfortable (2). It is just so nice to see people who aren’t just white. I feel like I belong. I am not different; and I am not othered. It is helpful to be able to share experiences and feel validated (3). I feel normal with people who all have similar backgrounds, circumstances, and upbringings. We don’t have to explain anything. Everybody just understands. Nobody has to teach someone else what something is. Everyone knows what you are talking about. We all understand and that's the biggest thing (2).

Getting mental health care and resources specifically targeted towards East Asian people is a challenge because there are not too many mental health experts that understand the East Asian experience, and definitely not enough for children. It is important that we have options available that we can relate to (4). When I was growing up, I was just different and there wasn’t the support. I never had the chance to have resources available for me. It was always hush hush. (Racism and the mental health impact) were never talked about. It was something that I just kept inside. I just learned to keep quiet. It was something that you just went through. (1).

But when I see resources and services from people who understand what I am going through, it helps so much. It is a gamechanger (7). So, this lack of suitable East Asian mental health experts is a barrier (3). In the healthcare system, I don’t want to (have to) use my voice to validate who I am, my identity, proving who I am, proving that I was born here (6). No matter how great of an ally a white person is, they cannot understand our experience. No matter how much I try to explain it to someone, they don’t understand what it's like, the pressures that you face as an immigrant child, the family dynamics. It is exhausting to try to explain this to someone. It is hard for white people to understand growing up in a ‘two cultural world’; it is just so different (3). The challenge of being East Asian and Canadian, and experiencing racism and the mental trauma of it. It is exhausting explaining to (mental health experts) the narrative behind a feeling that I am having when I say, *I don’t feel like I ever lived up to my parent's expectations*. It sounds stereotypical for me to say this and on the surface, non-East Asian people will say they get it. But they don’t get it unless they are in it. They just bypass all of the background history (4). It is hard to explain the context and sometimes it is traumatizing in its own to have to explain it (3).

There are many layers of understanding East Asian culture (1), our background (7), the immigrant experience (8), and experiences of racism. It is hard to make people understand that (1). For example, my parents have a saying, *bad news travels*. It is a Chinese saying. For Chinese people, at least my family and the majority of my friends’ family, we never discuss the bad. Everything's swept under the rug and you just deal with it (3). I understand where they are coming from. They are trying to protect you. The intentions are great. As first- or second-generation immigrant children, we face pressures to succeed. The immeasurable amount of pressure to succeed, to be seen a certain way, to not disappoint your family because they have given up so much; they have given you their life (3). The pressure to make your parents proud and be that perfect child, just like in the movie *Turning Red* (7). And within the Japanese culture, the belief of ‘losing face’ is a big thing (6). You don’t complain and you have to be respectful. You do everything for your family. And so, there is no such thing as mental health—it is called, suck it up. I was not able to talk about it with anybody as a child because my parents were also processing their experiences being immigrants to this country, the racism they probably felt. I was told to keep my head down, don’t cause trouble, and don’t cause a scene (4). If I have to explain this to someone who hasn’t lived it, who do not understand the context, they end up seeing my parents and family as villains (3). It is easy to point to East Asian cultures (4). But it is way more complex than that (3). So, we have to look through the East Asian lens rather than the white lens (2). Without the cultural context, a lot of our parents are monsters, but they are not (3). These experiences can’t be through a white lens or white experience. It will not resonate with me if it has been ‘whitewashed’. The thing with ‘whitewashing’ is this concept of having to explain myself or the East Asian experience, to explain aspects of my cultural background. Other people then tend to think, *oh, isn’t that quaint, isn’t that exotic* (2).

Understanding racism also plays a huge part of understanding the East Asian experience. I can’t really connect with someone in terms of racism if they’ve never experienced it. Growing up, racism pretty much defined almost everything that I did (4). It is hard because I look different from everyone else. People make comments about my nose, about my eyes. Being East Asian, I looked at myself and ask: *What is wrong with me? Why do I look like that? Why can’t I be white?* I felt I was ugly. I am very emotional just to bring it up because I don’t want my kids to go through that; go through the question of *what is wrong with me*. Even with Lunar New Year being such a big thing, I didn’t want to do it. I tried to hide it (1). All the East Asian things; like the stigma where East Asian people are known to own restaurants and grocery stores. My parents own a restaurant. Growing up, I had to work in the restaurant, and other kids didn’t do that. I remember thinking, *why am I so different, why do I have to work at the restaurant while other kids can do activities.* Growing up I never did activities or sports. I worked. I had to. I never had those opportunities. It was always push, push, push*.* (1). Recently, I spoke with my therapist about feeling really anxious about anti-Asian violence as it relates to COVID-19. They said I have to try to not focus on it. But I feel like if you had been in my position, if you had experienced racism the way I have growing up, they wouldn’t have been so quick to say that. I don’t want to explain to (mental health experts) what racism feels like. As a parent, I would never go to a parenting expert that did not have children. It's sort of that same rationale (4).

At the same time, I remember growing up that I talked to an Asian counselor who might as well have been a white person. The information that was coming back at me, the advice they gave, was not helpful in terms of dealing with East Asian cultural dynamics. I remember being really disappointed. I thought, *someone is going to finally understand me*. It didn’t work out that way and I just couldn’t understand why they couldn’t understand being an East Asian person themselves (5). So, the important thing is the relatability (6).

#### Composite Narrative 3: Unlearning, Breaking Barriers, and Storying Resistance

Composite narrative three reports on storylines: *breaking cycles*, and *culture as a source of strength*. *Breaking cycles* describes participant narratives seeking to unlearn their own biases towards mental health, make changes in the area of racism for their children, and amplify their voices and stories for the next generation. Participants use *culture as a source of strength* by neglecting the ‘model minority’ myth and releasing suffering as a source of strength and instead turning to their cultural roots of the family.

It is hard to talk about racism and mental health. It brings up all these emotions. It is hurtful. But I am okay and I am more concerned with my kids; I try not to let them experience what I went through. During my childhood, I didn’t want to be East Asian. I wanted to be like everyone else. I resented being East Asian. I wanted to be white. (1). I think I try really, really hard to not have my children go through what I went through in having to be in two different cultures and having to navigate that (5).

Now, we need to normalize the whole process of taking care of our mental health and speaking about our mental health. I never grew up with that, never had space for that. Now, I am (…) unlearning my own biases towards the mental health process (…) to be like *medications are okay*, *mental health support is okay*. It is a process because I have to write that in my mind first, then have to go and seek it out, go through the process when I am feeling uncomfortable with it but I do it for the benefit of my child. Then there is the process of having to then educate and walk my own parents and community through it. I have to deal with the triggers that come out of having to educate my parents. So, I have to do the work to teach myself and educate my parents. It is exhausting, but the end result is necessary (4). As East Asian parents, we have to create these opportunities for our children (7).

East Asian culture has a focus on family as a whole over the individual. If one person in the family is not doing well, we tend to hide it (3). We take care of each other. We have more of an internal system, be it physical illness or mental illness, even though we don’t talk about it as mental illness. If we see someone in our family is ill or suffering, we tend to take care of them in our household as opposed to thinking we need to go seek medical help (8). We do not have to let go of the family first mentality. But the family first mentality should also include healing (3), and taking time to grieve the vulnerabilities and the weaknesses (6). We also have to navigate the stereotype that East Asian people are supposed to be invisible, supposed to be submissive (5). To be obedient, compliant, and submissive (6). That East Asian people don’t need any help. That we have it easy, but we don’t (5). Being tough is not a strength and all it does is continue the trauma to the next generation (3). I am not the ‘model minority’; I want to break the stereotype (6). It^1^ should not override your own children's mental health and wellbeing. There are a lot of East Asian people in our generation in Canada that are determined to break these^2^ cycles. We are determined to get help for our children if they need it, and create a mentally healthier next generation. It is maddening and heartbreaking the pressure to keep up appearances (3).

As time goes on, I want my children to know there are services out there if they need them. I want them to know that there is support and that they should never feel ashamed of who they are. I really want them to kind of embrace their culture and being East Asian. There is more attention on racism now and that it is not acceptable. Where in the past, racism was acceptable. Nothing was done. There was no support. It is not right and we have to speak up. I feel that there is still racism. There will always be racism. It is just the way people are. But we need to hear our stories. To get them out there. To bring up our stories to educate. There will always be racism but hopefully that every little step we make will get better for the next generation (1).

## Discussion

This study sheds light on untold stories of East Asian parents and their perspectives of anti-racism strategies and engagement in research environments when developing KT resources. While the purpose of a narrative study is to explore the complexities and nuances of stories, rather than seek tangible solutions, this study offers critical openings into anti-racism strategies for future child mental health resource development which can have important consequences for the field of KT.

### Accessibility, Acceptability, and Representation

In congruence with other child health KT literature, participant narrative accounts described the need for resources to be accessible (Campbell et al., 2019; McBain et al., 2022; Thompson et al., 2020). Specifically, accessibility meant mental health resources being available in spaces such as their children's schools and being affordable. These findings are unsurprising given the extent of implementation science research dedicated to health equity in recent years (Baumann & Cabassa, 2020; Kelly et al., 2021; Woodward et al., 2021). Language barriers and translation services were highlighted as barriers to accessing child mental health resources, which aligns with other health KT literature focused on racialized or other systemically minoritized populations (Al-Sharifi et al., 2019; Poureslami et al., 2011). This study adds to the existing evidence base with specific East Asian parent accessibility needs. Participant narrative accounts highlighted that language barriers posed additional concerns within the area of mental health. Language barriers were compounded by the lack of vocabularies for parents to identify certain mental health concerns in their children. Participants explained that in East Asian cultural contexts, there may not be congruent terms or words in English to describe the same mental health symptoms or concerns. The awareness that all populations in Canada will not begin from the same cultural understanding is critically important when developing child mental health resources.

East Asian parents portrayed the need to ‘see’ themselves represented in the mental health resources. Their narrative accounts described that the process of representation may be the first step towards feeling safer and understood within the mental healthcare system. These findings align with research on the use of storybooks as a KT resource for parents of children diagnosed with croup where parents felt receiving information that mirrors similar situations and emotions to their own validates their experiences and normalized their experiences (Hartling et al., 2010; Scott et al., 2012). In the case of East Asian parents, representation may become an entry point to which the perceived acceptability of their child's mental health resources is influenced (Sekhon et al., 2017). These insights highlight the need to utilize approaches to KT resources that hold nuance for language, vocabulary, and representation needs. Furthermore, foundations of cultural safety offer strategies to mitigate these barriers. Cultural safety aims to acknowledge barriers to equitable and quality care by reflecting on differences and power relations (Curtis et al., 2019; Papps & Ramsden, 1996). The application of cultural safety provides researchers considerations to examine their biases in KT resource development, and therefore, provide solutions to the barriers for accessing mental health resources identified by the participants.

### Complexities of Engaging East Asian Parents in Research

#### Whiteness, Racial Trauma, and Intentions

While representation acted as an initial barrier in seeking child mental health resources, East Asian parents’ narratives exposed tensions with solely focusing on representation. Participants storied that despite having the aforementioned accessibility barriers addressed, some East Asian parents would still not access child mental health resources. Existing literature exploring Asian diasporas’ mental health resource utilization has identified cultural or individual reasons, such as mental health stigma, as a barrier to seeking resources (Abdullah & Brown, 2011; Fung et al., 2022; Fung & Wong, 2007; Kim & Lee, 2014; Sadavoy et al., 2004). This study extends this area of inquiry by highlighting participants denied utilizing mental health resources because in many cases, the resources did not appreciate the complexities facing East Asian peoples. Participants storied how a lack of fully grasping East Asian experiences may fail to attend to the unspoken rules, the indescribable understandings, and the ways of knowing and being that must be lived through rather than explained and interpreted.

Critically, participants’ experiences of racism and mental health remained varied given the “many layers of understanding East Asian culture” (composite narrative 2). These ‘layers’ of understanding can be further analysed through Black feminist thought and activism. Patricia Hill Collins’ notion of intersectionality within the matrix of domination fosters a critical understanding with how interconnected systems of oppression, such as racism, classism, and sexism, operate within historical and social structures in which power is perpetuated (Collins, 2000). Experiences and social reality, therefore, vary as a result of individuals’ intersecting oppressions and relation to power (Collins, 2000). When taking an anti-essentialist approach for understanding the individual experience (Delgado & Stefancic, 2013, 2017), the participant narratives can be further understood by being positioned within a matrix of oppression that shape their experiences (Collins, 2000). For example, the narrative of an East Asian parent not being understood by an Asian counselor (composite narrative 2) can in part be explained by the multiple axes both the East Asian parent and Asian counselor are positioned within (e.g., class, gender) to inform their interaction. These findings are imperative for the future of child mental health KT by illuminating the indescribable components of the East Asian experiences, and importantly, how these experiences are layered, situated, and operate within matrices of oppression. Therefore, future resources require moving beyond the notion of representation (i.e., simply ‘seeing’ themselves in resources) by first, attending to the complexities of the East Asian experience through the multiple unspoken ways of knowing and being, and second, exploring how the indescribable East Asian experiences operate within intersecting oppressions.

Participants further described that whiteness is the taken-for-granted perspective in knowledge creation and the default audience for developing resources. These findings make sense given the extent of covert racism within the theoretical foundations of nursing and other healthcare disciplines, and the egalitarian values within the Canadian context that allows knowledge subtly sustaining white supremacy (Louie-Poon et al., 2021). As explained elsewhere, a limited perspective of whiteness will produce knowledge only within the confines of whiteness (Louie-Poon et al., 2021), further propagating the goals of white supremacy through KT resources. This study extends this knowledge by highlighting the exhaustion and traumatizing nature of explaining and re-explaining stories to researchers or healthcare professionals. These findings are congruent with CRT which explain that racism works by re-storying racialize groups through whiteness that maintains their subordinate racial positioning (Delgado & Stefancic, 2017; Liu et al., 2019; Liu et al., 2023; Solórzano & Yosso, 2002). Moreover, given the pervasiveness of anti-Asian racism within Canadian society, the persistent exposure to painful encounters of being re-storied through whiteness is consistent with the literature on racial trauma (Carter & Muchow, 2017; Cénat, 2023; Liu et al., 2019).

These findings storying the impact of exhaustion and racial trauma provide critical insights and lessons for KT approaches particularly when engaging with East Asian populations. Aligned with concepts of transcending whiteness and moving towards solidarity for racialized voices (Liu et al., 2023; Solórzano & Yosso, 2002), participants outlined the tensions and implications with notions of allyship and partnership. East Asian parent narratives described that despite ‘good intentions’, a person who has not lived through their situations may lack the capacity to understand their situations fully. As storied by participants, this lack of understanding of the complexities of the East Asian situation has consequences when researchers or health professionals use this limited standpoint to develop evidence, thus (in)advertently further reinforcing harmful stereotypes. These findings are congruent with the existing anti-racism recommendation within the implementation science literature calling for the need to critically reflect if evidence adequately reflects lived experiences of racism (Shelton, Adsul, Oh, Moise, et al., 2021).

Within the context of our findings, the lack of contextual and situational knowledge of the East Asian experience inhibits the accurate interpretation of events. For example, participants highlighted the immeasurable pressures to ‘succeed’, not cause ‘trouble’ or ‘complain’, and not address their mental health concerns. These examples shed light on the ‘model minority’ myth; an inaccurate stereotype tokenizing Asian diasporas as a hardworking, successful group of people when in comparison to other racialized populations (Pon, 2000). Moreover, the ‘model minority’ myth uses the incorrect perception that Asian diasporas are unaffected by racialization, and thereby strategically silencing experiences of racism even in the face of individual and collective anti-Asian violence (Pon, 2000; Tessler et al., 2020). In the absence of critically reflecting on East Asian parents’ narratives within a comprehensive understanding of the ‘model minority’ myth and how this impacts East Asian communities, researchers risk misinterpreting East Asian experiences or further reinforcing this myth. As participant narrative accounts outline, researchers may misconstrue their families as ‘monsters’ or ‘villains’ in relation to their experiences as a result of the ‘model minority’ myth. This has significant implications for the development of child mental health KT resources. Particularly, these misinterpretations, which may be re-storied through whiteness to blame East Asian cultures or place East Asian diasporas in a negative standpoint, reproduce white supremacy narratives by not implicating the structural violence causing the harm. In this scenario, without interrogating the ‘model minority’ myth rooted in white supremacy ideologies and the reasons why such narratives arise in society, these (un)intentional, yet racially biased, understandings become the standpoint from which child mental health KT resources may be developed. This example showcases why it is imperative for researchers to remain critically reflective of the evidence informing the development of KT resources (Shelton, Adsul, Oh, Moise, et al., 2021), and the importance of contextual knowledge from the standpoint of East Asian perspectives.

#### Epistemic Racism and Silencing East Asian Voices

Furthermore, these findings align with the philosophical tenets of CRT that promote agency of racialized voices (Delgado & Stefancic, 2017; Solórzano & Yosso, 2002). Specifically, the participant narratives do not suggest voicelessness, rather warrant a need to resist and dismantle the very system that situate them as voiceless (Spivak, 2010). This notion of seeking to amplify racialized standpoints in pursuit of unearthing social injustices is highlighted by the CRT tenet of counter-narratives. CRT scholarship notes that racialized populations have the competence to utilize their voices on racism through counter-narratives, and thus, providing a richer understanding of their own realities (Delgado & Stefancic, 2013, 2017; Solórzano & Yosso, 2002). Interestingly, in the participants’ development of their counter-narratives through this study, they highlighted the value for East Asian parents to speak about racism through a counter-narrative in research contexts. These findings are closely linked to Spivak's (2010) postcolonial feminist theory of the subaltern. In the context of Spivak (2010), subaltern refers to those without power. As Spivak (2010) asserts, western academic knowledge often commits epistemic violence by speaking for and on behalf of subalterns. If applying Spivak's (2010) notions to our findings, subalterns may be the East Asian ‘patient partners’ who continue in the subordinate position when compared to the researchers who continue holding institutional power. The voices of the East Asian parent partners are represented through the researchers as a way for East Asian people to *speak* in research. While this can be seen as an attempt to liberate the voices of East Asian people as a partner within the academic institution, this very process enacts epistemic racism, and calls into question the notion of agency as highlighted in CRT scholarship.

East Asian peoples as research ‘partners’ have always existed outside the realm of power within research without holding true power. As members of society, East Asian parents are used as tools of white supremacy, where their Asianness permits them as holding privilege by their closeness to whiteness, while always being threatened with notions of being a ‘foreigner’ (Ang, 2011; Gover et al., 2020; Nguyen et al., 2019). Given that research cannot be extracted from the larger conditions of society (Louie-Poon et al., 2021), this threat was layered in our findings when East Asian parents shared stories of navigating their racial positioning and silenced perspective (composite narrative 2). These findings make sense within Spivak's (2010) notion of romanticizing the ‘other’—the idea that dominant cultures perpetuate power imbalances through stereotypes of systemically minoritized populations. This has significant implications for understanding patient engagement given that East Asian parents may not have full agency under current conceptualization of ‘power sharing’ in research (Nguyen et al., 2020) due to the larger conditions of white supremacy, thus limiting their ability to become true research ‘partners’.

Our participant narratives provide KT research with critical insights when engaging *with* patient partners. In the pursuit of amplifying East Asian parent voices, our participants storied how these processes have inflicted more harm by inviting them into systems without researchers, mental health professionals, or institutions critically self-reflecting about the violence espoused from these spaces. Instead, East Asian parents have been expected to adapt to a system built on the foundations of white supremacy. These findings align with literature critiquing traditional patient engagement strategies that lack an intersectional understanding of how systemic barriers, such as racism, sexism, colonialism, ableism, and heterosexism, prevent individuals from meaningful inclusion and engagement (Shimmin et al., 2017). As stated by Shimmin et al. (2017), without addressing these complexities and contexts of systemically oppressed individuals, patient engagement approaches will remain problematic by lacking an understanding of the multitude ways (i.e., unable to access, stigma, unsafety) individuals may or may not engage with health research or the healthcare system. Our findings further contextualize this area of inquiry by highlighting that this process impacts East Asian parents by producing harm, racial trauma, and violence on their mental wellbeing through epistemic racism and a lack of agency. Nonetheless, our participants offered insights into ways these narratives may shift for future research.

### Toward a Solution

#### Inviting East Asian Standpoint Epistemology and Counter-Spaces

Based on our findings, beginning with the social location of East Asian parents may amplify their socially relevant issues. These findings make sense when we situate these findings within feminist standpoint theory (FST), which raises the epistemological question of how power shapes knowledge (Harding, 1997). According to FST, knowledge development cannot be decontextualized from larger influencing forces of sociocultural structures on knowledge, nor the social identity of a knower. Thus, one's social relation to power acts as both an enabler and a limiter of knowledge (Harding, 1997; Swigonski, 1994). FST claims that oppressed groups experience a different reality as a result of their marginalization. To adequately navigate society, therefore, oppressed groups obtain an awareness of both the dominant worldview and their own, affording them with less partial and distorted knowledge. This standpoint of oppressed groups offers a more complete account of reality, which holds an epistemological advantage (Harding, 1997; Swigonski, 1994; van Wormer, 2009), suggesting that inquiry concerning oppressed groups ought to begin with their standpoint. Therefore, developing knowledge through an East Asian standpoint epistemology may permit a more comprehensive understanding of the complexities of East Asian narratives without diluting their stories. For example, an East Asian standpoint epistemology may develop information on the concept of ‘losing face’ from the East Asian standpoint. This approach may center the complexities of East Asian lived experiences of the pressures to honor the family name, while avoiding reinforcing harmful stereotypes of East Asian cultures or losing the historical, social, cultural, and racial context of the narratives. Based on our findings and rooted in FST (Harding, 1997), the East Asian standpoint epistemology redistributes power towards East Asian peoples as a site and location of knowledge building. These findings are congruent with literature from the development of critical race methodologies for research practices in the education discipline (Solórzano & Yosso, 2002). Solórzano and Yosso (2002) highlight how telling stories through the standpoint of racialized people works to amplify groups who are epistemologically silenced.

The participants further situated their East Asian standpoints within the context of a “shared experience” (composite narrative 2). Participants story the tensions of navigating the mental healthcare spaces that are predominately white, where their East Asian experiences were overlooked. Within the context of CRT, these findings are further explained given that master-narratives are strategically utilized to produce and reinforce whiteness (Delgado & Stefancic, 2013, 2017; Solórzano & Yosso, 2002). Existing literature within CRT has negotiated these spaces with racial counter-spaces, or spaces where racialized populations navigate healing and solidarity within the shared experiences of their racial positioning beyond the white gaze or space (Liu et al., 2023; Solórzano & Yosso, 2002). Rooted in this understanding, participants storied the critical importance of an East Asian counter-space as a way to situate and facilitate the centrality of their East Asian standpoint epistemologies.

These insights may be useful for the field of KT by developing child mental health resources through an East Asian standpoint epistemology (i.e., what knowledge is developed) and within an East Asian counter-space (i.e., how and where the knowledge is developed). It is important to note that while our study is situated within the field of child mental health KT, these findings may extend to other contexts beyond child mental health and may be applied to research processes more broadly given the institutionalized and systemic nature of racism. Based on our participant narratives, an East Asian standpoint epistemology may benefit East Asian parents by not having their narratives re-storied through whiteness or requiring their explanation of East Asian standpoints, both of which participants described as traumatizing. Furthermore, developing this knowledge within an East Asian counter-space may overcome the participant narratives of exhaustion and racial trauma by ensuring their safety, lived realities, and counter-stories are honored, centered, and understood. These findings align with the emerging literature focused on equity-oriented, anti-racist, and user-centered designs (Baumann & Cabassa, 2020; Shelton, Adsul, Oh, Moise, et al., 2021), and critically analyzing whose voices are heard, valued, and reflecting on who benefits within research spaces (Shelton, Adsul, Oh, Moise, et al., 2021). We understand these approaches to be different from the frameworks historically centered in KT and iKT given the philosophical underpinnings from which knowledge is conceptualized and developed that begins with and amplifies non-dominant ways of knowing while decentering whiteness. This remains particularly novel from existing iKT approaches given the emphasis to avoid adding voices for a diversity of perspective; rather, it seeks to shift the underpinning philosophical standpoint from which inquiry begins. In this lens, a structural reimagining with how we enter, and subsequently, engage in research processes attends to the roots of white supremacy for East Asian parents in research contexts. To further contextualize this East Asian standpoint epistemology and counter-spaces within Shimmin et al.'s (2017) proposed trauma-informed intersectional analysis, these findings align with the practice of continuously examining contexts of power and negotiating social locations. Particularly, East Asian standpoint epistemology is congruent with determining how the perspectives of people with lived experiences are centered in research (e.g., understanding roles, including people with lived experiences, avoiding reinforcing existing stereotypes), while an East Asian counter-space supports the need to ensure the safety (e.g., interpersonal setting, physical spaces) of all members involved in research (Shimmin et al., 2017).

#### Shifting Narratives to Recenter the Counter-Narratives

Embedding East Asian standpoint epistemology and counter-spaces as the philosophical underpinnings for child mental health KT may re-envision how we amplify and develop resources ‘for us, by us’. This idea is not novel within the area of anti-racism and social justice, and has been well-developed by social activist and postcolonial and critical race theorists when critiquing issues of power and whiteness (Delgado & Stefancic, 2013, 2017; Liu et al., 2019, 2022, 2023; Patton & Catching, 2009; Spivak, 2006, 2010). More recently, this concept has been illuminated within the field of patient engagement by shifting the lens towards a social justice framing (Shimmin et al., 2017). Within the context of this study, ‘for us, by us’ provides us with new dimensions and solutions for East Asian parents. Particularly, beginning resource development with East Asian standpoint epistemology and from an East Asian counter-space allows East Asian parents the agency to story their counter-narratives and actively challenge the master narratives often constructed to sustain whiteness (Delgado & Stefancic, 2017; Liu et al., 2019; Liu et al., 2023). It calls into question what it means to commit to the process of unlearning whiteness and advocate for anti-racism principles in KT: *Do we enact silencing of East Asian parents by inadvertently ‘speaking for’ patient partners? How do partnerships within KT and iKT re-story East Asian people through whiteness and cause the furthering of racial trauma?* Researchers may remain cognizant of these factors through the use of narrative based strategies when engaging with East Asian partners within research to provide space for the complexity within lived experiences of East Asian communities, prioritize ongoing rapport, and implementing storytelling methods as a means of understanding, communication, and knowledge sharing.

Congruent with the points raised above that were critical of the notion of voicelessness, East Asian parents were reclaiming their stories beyond the white gaze through their counter-narratives (Delgado & Stefancic, 2017; Liu et al., 2023; Solórzano & Yosso, 2002). Contrary to mainstream narratives, East Asian parents in our study storied narratives of already ‘doing the work’ of unlearning their internalized racism, re-learning that mental health help and wellness is necessary, that their cultural focus on family first ought to include healing, and resisting pressures of the ‘model minority’ stereotypes (composite narrative 3). These often-untold stories demonstrate their resistance in the face of white supremacy. Our findings differ from the narratives found in previous literature from the past decade in the American context stating that Asian diasporas may internalize ‘model minority’ stereotypes that may negatively impact their mental health (Gupta et al., 2011; Kim & Lee, 2014). However, these narratives of resisting racism are supported in a recent study from the American context. Liu et al. (2022) found that despite participants being subjected to ‘foreigner’ stereotypes that escalated during the COVID-19 pandemic, participants utilized their communities and resilience to counteract racism. Our findings in the context of this previous literature suggest the potential shift in narratives and how East Asian parents have negotiated and resisted epistemic silence within their counter-spaces (Liu et al., 2023).

### Future Considerations and Limitations

These findings advance the areas of patient-orientated and KT research by providing anti-racism foundations through the perspective of East Asian parents—a often historically minoritized community in research. Specifically, philosophical openings and critical dialogue are provided to address racism within child mental health KT. Researchers need to scrutinize what resources are currently being offered in terms of child mental health resources, and critically question if adapting previous resources or redeveloping new ones is necessary. Drawing from abolitionist thought and a radical philosophy for the nursing discipline (Hopkins-Walsh et al., 2023), attempting to reform a white supremacist system will never eliminate racism given its unjust foundation of structural violence and exploitation. Similarly, adapting resources from the lens of whiteness and from a racist context will not eliminate racism, and may only solve the accessibility barriers as explained in composite narrative 1, without attending to the other critical factors of subalternity, epistemic racism, silencing, and racial trauma. Under a social justice purview, if nurses are committed to an anti-racist principle with East Asian standpoint epistemologies, counter-spaces, and counter-narratives at the forefront, the redevelopment of child mental health resources is necessary that centers East Asian complexities led by East Asian peoples. Based on our study insights, future research may consider addressing structural injustices that often preclude East Asian perspectives within academic institutions and re-envision future locations and contexts of knowledge building. This may be an entry-point for reimagining child mental health resources for East Asian populations with East Asian parents; *for us, by us*.

There are some limitations to this study. First, all participant interview data were used for the development of composite narratives; however, one participant's second interview recording data were lost due to technical issues during the recording saving process. Therefore, a total of 16 interviews were conducted with 8 participants (i.e., two interviews per participant), with 15 interviews included for the development of composite narratives. Detailed reflexive notes were documented immediately following the participant's interview where recorded data were lost and used for the analysis and interpretation of data. Verbatim quotes for the composite narratives were only integrated from this participant's first interview, potentially impacting our findings transferability. However, our study weaves together multiple direct quotes across all participants within the composite narratives to support the storylines and strengthen study rigor. Given the nature of online interviews and potential for technical issues such as recording, future studies may consider utilizing strategies with two recording functions to prevent data loss and enhance study rigor. Second, while the study utilized purposive sampling to attempt to achieve a sample with various East Asian perspectives, no participants self-identified as Korean. This may have limited the diverse variation in anti-Asian racism perspectives in our study findings. Future studies with a focus on uncovering anti-Asian racism from the perspective of East Asian parents may tailor their recruitment strategy to specific organizations or community centers, rather than broadly recruiting through anti-racism and child health organizations.

## Conclusion

This study brings new perspectives and dialogue into the field of child mental health KT. Through the narratives of 8 East Asian parents, opportunities for alternative possibilities when developing resources with East Asian diasporas are opened. This study calls into question the conditions within KT that (in)advertently reinforce harmful stereotypes of East Asian diasporas as a weapon of epistemic racism. We also explored how covert whiteness framed within the idea of partnership may be a form of strategic silencing. With these new dimensions in KT illuminated, the authors explore anti-racism strategies in KT through East Asian standpoint epistemology, counter-spaces, and counter-narratives. These insights open future critical dialogues into power and anti-racism within child mental health KT.

## Supplemental Material

sj-docx-1-cjn-10.1177_08445621251322552 - Supplemental material for A Narrative Inquiry of East Asian Parents and Mental Health in Canada: Critical Openings for Anti-Racism Strategies in Knowledge Translation Supplemental material, sj-docx-1-cjn-10.1177_08445621251322552 for A Narrative Inquiry of East Asian Parents and Mental Health in Canada: Critical Openings for Anti-Racism Strategies in Knowledge Translation by Samantha Louie-Poon, Solina Richter, Diane Kunyk and Shannon D. Scott in Canadian Journal of Nursing Research

## Notes

1. Referring to the ‘model minority’ myth.

2. Referring to breaking the cycle of family first mentality that does not include healing and upholding appearances of the ‘model minority’.
