# Supplementary material for: A Narrative Inquiry of East Asian Parents and Mental Health in Canada: Critical Openings for Anti-Racism Strategies in Knowledge Translation
Source: Can J Nurs Res. 2025 Mar 17;57(1):8–23. doi: 10.1177/08445621251322552 (PMC11967094; doi:10.1177/08445621251322552)
Supplement: sj-docx-1-cjn-10.1177_08445621251322552 - Supplemental material for A Narrative Inquiry of East Asian Parents and Mental Health in Canada: Critical Openings for Anti-Racism Strategies in Knowledge Translation [file sj-docx-1-cjn-10.1177_08445621251322552.docx]

# Supplemental File A: Participant Quotes Exemplars

| **Composite narrative 1:** Storying issues of access within child mental health KT | **Storyline**:  Availability and affordability | **Selected Verbatim Quotes:**   - If mental healthcare were more affordable, it would be more accessible to East Asian communities (4). - There is no centralized system for East Asian people to go to (3). |
| --- | --- | --- |
|  | **Storyline**:  Language and vocabulary (i.e., terminologies) barriers | - I can do the research, but if there are no websites that translate back to the language I prefer to operate in, it is very frustrating. I don’t have the energy supply to really look for multiple resources or services that offer translation. Google translate has word limits and limits on how much can be translated. It is taxing to use, and even though these are options, somebody who is less digital savvy, in a new environment, and in more social economic stress will have a shorter of a fuse or attention span to go through translating word by word, phrase by phrase (8). - It would feel more relatable if there was a sheet or resource with certain East Asian experiences. Right now, a family member is in desperate need of some mental health help. But they will not seek it out despite having all the current resources available to them (3). |
|  | **Storyline**:  Lack of representation | - So, it would help to have more representation and have more options available with a deep understanding of our culture and background. When there is more representation, it is a sense of belonging. It is almost like visceral—I feel my body relax. It is a comfort (4). |
| **Composite narrative 2:** Seeking understanding and solidarity for the East Asian story | **Storyline**:  Issues of representation: power and whiteness | - No matter how great of an ally a white person is, they cannot understand our experience. No matter how much I try to explain it to someone, they don’t understand what it’s like, the pressures that you face as an immigrant child, the family dynamics. It is exhausting to try to explain this to someone. It is hard for white people to understand growing up in a ‘two cultural world’; it is just so different (3). - The challenge of being East Asian and Canadian, and experiencing racism and the mental trauma of it. It is exhausting explaining to (mental health experts) the narrative behind a feeling that I am having when I say, *I don’t feel like I ever lived up to my parent’s expectations*. It sounds stereotypical for me to say this and on the surface, non-East Asian people will say they get it. But they don’t get it unless they are in it. They just bypass all of the background history (4). |
|  | **Storyline**:  East Asian standpoint epistemology | - It is about knowing the indescribable, non-language understanding of how things are. The unspoken rules. The unwritten background. I can try to explain this. And others can try to understand it but they will only know to the degree of the stereotypical ideas; the things that they see on TV (7). - If I have to explain this to someone who hasn’t lived it, who do not understand the context, they end up seeing my parents and family as villains (3). - These experiences can’t be through a white lens or white experience. It will not resonate with me if it has been ‘whitewashed’. The thing with ‘whitewashing’ is this concept of having to explain myself or the East Asian experience, to explain aspects of my cultural background. Other people then tend to think, *oh, isn’t that quaint, isn’t that exotic* (2). |
| **Composite narrative 3:** Unlearning, breaking barriers, and storying resistance | **Storyline**:  Breaking cycles | - As time goes on, I want my children to know there are services out there if they need them. I want them to know that there is support and that they should never feel ashamed of who they are. I really want them to kind of embrace their culture and being East Asian. There is more attention on racism now and that it is not acceptable. Where in the past, racism was acceptable. Nothing was done. There was no support. It is not right and we have to speak up. I feel that there is still racism. There will always be racism. It is just the way people are. But we need to hear our stories. To get them out there. To bring up our stories to educate. There will always be racism but hopefully that every little step we make will get better for the next generation (1). |
|  | **Storyline**:  Culture as a source of strength | - We do not have to let go of the family first mentality. But the family first mentality should also include healing (3), - Being tough is not a strength and all it does is continue the trauma to the next generation (3). |
